# Comparative characterization of two intracellular Ca^2+^-release channels from the red flour beetle, *Tribolium castaneum*

**DOI:** 10.1038/srep06702

**Published:** 2014-10-21

**Authors:** Yaping Liu, Chengjun Li, Jingkun Gao, Wenlong Wang, Li Huang, Xuezhu Guo, Bin Li, Jianjun Wang

**Affiliations:** 1 College of Horticulture and Plant Protection, Yangzhou University, Yangzhou, 225009, China; 2 Jiangsu Key Laboratory for Biodiversity and Biotechnology, College of Life Sciences, Nanjing Normal University, Nanjing, China

## Abstract

Ryanodine receptors (RyRs) and inositol 1,4,5-trisphosphate receptors (IP_3_Rs) are members of a family of tetrameric intracellular Ca^2+^-release channels (CRCs). While it is well known in mammals that RyRs and IP_3_Rs modulate multiple physiological processes, the roles of these two CRCs in the development and physiology of insects remain poorly understood. In this study, we cloned and functionally characterized RyR and IP_3_R cDNAs (named *TcRyR* and *TcIP_3_R*) from the red flour beetle, *Tribolium castaneum*. The composite *TcRyR* gene contains an ORF of 15,285 bp encoding a protein of 5,094 amino acid residues. The *TcIP_3_R* contains an 8,175 bp ORF encoding a protein of 2,724 amino acids. Expression analysis of *TcRyR* and *TcIP_3_R* revealed significant differences in mRNA expression levels among *T. castaneum* during different developmental stages. When the transcript levels of *TcRyR* were suppressed by RNA interference (RNAi), an abnormal folding of the adult hind wings was observed, while the RNAi-mediated knockdown of *TcIP_3_R* resulted in defective larval–pupal and pupal–adult metamorphosis. These results suggested that *TcRyR* is required for muscle excitation-contraction (E-C) coupling in *T. castaneum*, and that calcium release via *IP_3_R* might play an important role in regulating ecdysone synthesis and release during molting and metamorphosis in insects.

Calcium (Ca^2+^) is a key second messenger that plays important physiological roles in various cells. There are two main Ca^2+^ mobilizing systems in eukaryotic organisms including Ca^2+^ influx through the plasma membrane and Ca^2+^ release from internal stores. Ryanodine receptors (RyRs) and inositol 1,4,5-trisphosphate receptors (IP_3_Rs) are large tetrameric intracellular Ca^2+^-release channels (CRCs) located in the endo/sarcoplasmic reticulum (ER/SR) of cells. An increasing number of both RyR and IP_3_R functional genes have been identified in a variety of multicellular eukaryotes ranging from *Caenorhabditis elegans* to humans^1^, and recently, putative RyR/IP_3_R homologs have also been identified in unicellular organisms^2^,^3^. In mammals, three isoforms of RyRs (RyR1, RyR2 and RyR3) and IP_3_Rs (IP_3_R1, IP_3_R2 and IP_3_R3) have been identified, which are encoded by separate genes and show distinct cellular distribution patterns. While the IP_3_Rs are approximately half the size of the RyRs, these two receptors show similarities in their regulation, and a recent study indicated that RyRs and IP_3_Rs have co-evolved from an ancestral unicellular RyR/IP_3_R^1^.

In contrast to mammals, only one of each RyR (*DmRyR*) and IP_3_R (*DmIP_3_R*) gene was identified in *Drosophila* *melanogaster*^4^,^5^,^6^, which showed approximately 45% and 60% amino acid identity with the three mammalian RyRs and IP_3_Rs, respectively. Compared with IP_3_Rs, insect RyRs have attracted increasing attention due to the discovery of diamide insecticides including the compounds flubendiamide, chlorantraniliprole (Rynaxypyr^TM^) and cyantraniliprole (Cyazypyr™)^7^,^8^. Functional expression studies of the recombinant silkworm RyR (*sRyR*) in HEK293 cells have suggested that the insecticide flubendiamide is mainly incorporated into the transmembrane domains (residues 4111-5084) of sRyR^9^. Recently, a short segment of the C-terminus transmembrane region of DmRyR (residues 4610-4655) was found to be critical to diamide insecticide sensitivity^10^. Additionally, it was reported that high levels of diamide cross-resistance in *Plutella xylostella* are associated with a target-site mutation (G4946E) in the COOH-terminal membrane-spanning domain of the RyR^11^. Beyond the recent characterization of RyRs in moths and fruit flies, little molecular characterization of insect IP_3_Rs has been performed.

It is well known in mammals that RyRs and IP_3_Rs modulate a wide variety of Ca^2+^-dependent physiological processes^1^,^12^. However, information about the physiological processes affected by their function in insects is still limited. In the present study, we cloned RyR and IP_3_R cDNAs (named as *TcRyR* and *TcIP_3_R*) from the red flour beetle, *Tribolium castaneum*. We report the expression patterns of the *TcRyR* and *TcIP_3_R* transcripts. We also explored the roles of these two CRC genes in the development and physiology of *T. castaneum* by in vivo RNA interference (RNAi).

## Results

### cDNA Cloning and characterization of *TcRyR* and *TcIP_3_R* in *Tribolium castaneum*

**Figure 1:**
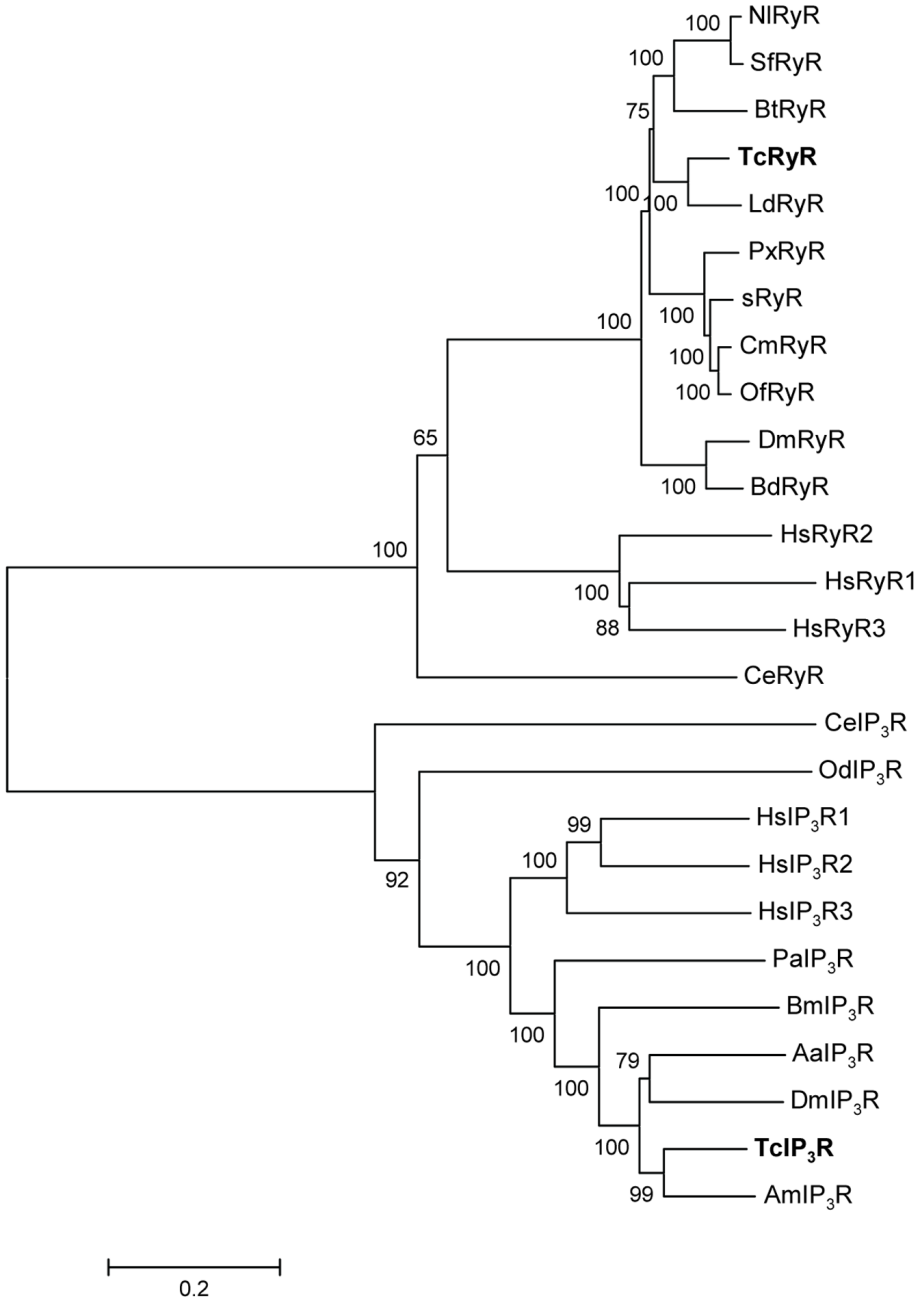
Phylogenetic tree of the RyR and IP_3_R families. A multiple alignment of TcRyR and TcIP_3_R amino acid sequences with representative RyR and IP_3_R isoforms was performed and used as the input for phylogenetic analysis. The Neighbor-joining tree was generated using MEGA5 with 1000 bootstrapping. RyR sequences are obtained from the following GenBank entries: DJ085056 for *Bombyx mori* (sRyR); AET09964 for *Plutella xylostella* (PxRyR); BAA41471 for *Drosophila melanogaster* (DmRyR); AFK84957 for *Bemisia tabaci* (BtRyR); JQ799046 for *Cnaphalocrocis medinalis* (CmRyR); AHW99829 for *Sogatella furcifera* (SfRyR); AHW99830 for *Leptinotarsa decemlineata* (LdRyR); AHY02115 for *Bactrocera dorsalis* (BdRyR); AGH68757 for *Ostrinia furnacalis* (OfRyR); KF306296 for *Nilaparvata lugens* (NlRyR); BAA08309 for *Caenorhabditis elegans* (CeRyR); NM__-_000540 for *Homo sapiens* RyR1 (HsRyR1); NM_001035 for *Homo sapiens* RyR2 (HsRyR2); NM_001243996 for *Homo sapiens* RyR3 (HsRyR3). IP_3_R sequences are obtained from the following GenBank entries: AAN13240 for *D. melanogaster* (DmIP_3_R); EAT33105 for *Aedes aegypti*(AaIP_3_R); XP_004923625 for *B. mori* (BmIP_3_R); XP_006564780 for *Apis mellifera* (AmIP_3_R); CCD63765 for *C. elegans* (CeRyR); AAT47836 for *Oikopleura dioica*(OdIP_3_R); AAC61691 for *Panulirus argus* (PaIP_3_R); NP_001161744 for *Homo sapiens* IP_3_R1 (HsIP_3_R1); NP_002214 for *Homo sapiens* IP_3_R2 (HsIP_3_R2); NP_002215 for *Homo sapiens* IP_3_R3 (HsIP_3_R3).

RT-PCR was used to amplify the entire coding sequences of the RyR and IP_3_R cDNAs from *T. castaneum.* A total of 12 and 6 overlapping cDNA fragments were obtained for *TcRyR and TcIP_3_R,* respectively (Table 1). Compilation of the cDNA clones resulted in a 15,308 bp contiguous sequence containing a 15,285 bp ORF for *TcRyR* and an 8,231 bp contiguous sequence containing an 8,175 bp ORF for *TcIP_3_R*. Amino acid sequence alignments showed that the encoded 5,094 amino acid residues of *TcRyR* and 2,724 amino acid residues of *TcIP_3_R* share 78% and 70% overall amino acid identity with the *D.* *melanogaster* DmRyR and DmIP_3_R, respectively. The overall amino acid identities of TcRyR with its human homologues, HsRyR1, HsRyR2 and HsRyR3, were 44%, 46% and 44%, respectively, while identities of TcIP_3_R with human homologues HsIP_3_R1, HsIP_3_R2 and HsIP_3_R3 were 61%, 58% and 53%, respectively. Phylogenetic analyses were consistent with these proteins representing RyR and IP_3_R homologues, respectively (Fig. 1).

The sequence alignments also revealed the conservation of critical amino acid residues within TcRyR and TcIP_3_R. For example, a glutamate residue proposed to be involved in the Ca^2+^ sensitivity of the rabbit RyR3 (E^3885^)^13^ and RyR1 (E^4032^)^14^ was detected in TcRyR (E^4140^). Additionally, residues corresponding to I^4897^, R^4913^, and D^4917^ of the rabbit RyR1, which were recently shown to play an important role in the activity and conductance of the Ca^2+^release channel^15^, were also conserved in TcRyR (I^4950^, R^4966^, D^4970^). Eleven amino acid residues known to be important for the strict recognition of IP_3_ within the IP_3_-binding core domain of the mouse IP_3_R1^16^ were conserved in TcIP_3_R (R^267^, T^268^, T^269^, G^270^, R^271^, R^496^, K^500^, R^503^, Y^560^, R^561^, K^562^). Seven residues in the NH_2_-terminal suppression domain of the mouse IP_3_R1 critical for the suppression of IP_3_ binding^17^ were also found in TcIP_3_R (L^31^, L^33^, V^34^, D^35^, R^37^, R^55^, K^128^).

**Figure 2:**
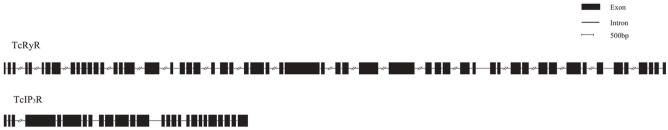
Schematic diagrams of the genomic organization for *TcRyR* and *TcIP_3_R.*

**Figure 3:**
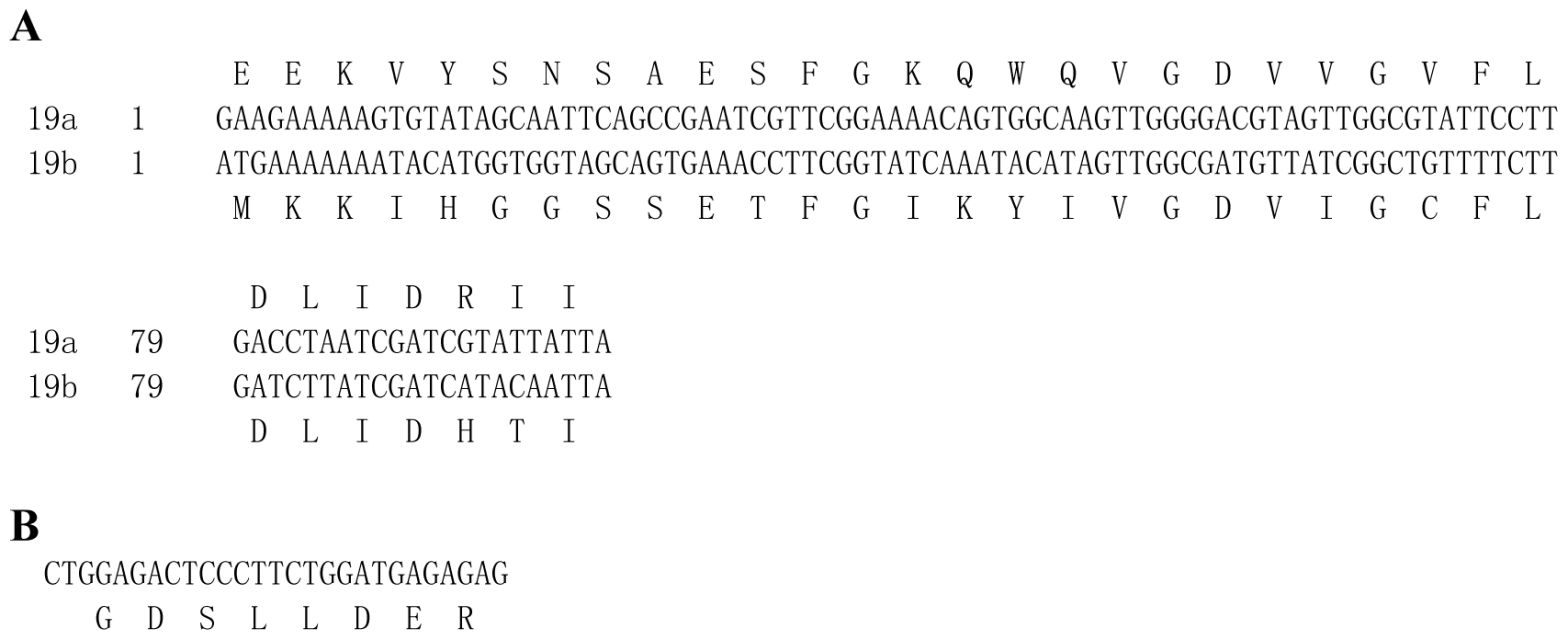
Nucleotide and inferred amino acid sequences of alternative exons in *TcRyR* and *TcIP_3_R*.

The genomic structures of *TcRyR* and *TcIP_3_R* were predicted by comparing the composite cDNA sequences with the genomic sequences retrieved from contigs in the whole genome shotgun release for *T. castaneum*^18^ (Fig. 2). The *TcRyR* comprises 55 exons ranging in size from 54 bp to 1462 bp including a pair of mutually exclusive exons (19a/19b, Fig. 3A), which were confirmed by multiple cDNA clone sequence alignment and were conserved in other insect RyRs^6^,^19^,^20^. The *TcIP_3_R* was split into 26 exons ranging in size from 71bp to 1269 bp. The 5′ donor and 3′ acceptor site sequences in both *TcRyR* and *TcIP_3_R* were in agreement with the GT/AG consensus sequence, except the 5′ donor sequence (GC) for intron 7 in *TcRyR*. Additionally, the alignment of multiple cDNA clone sequences also revealed one alternative splice site in *TcIP_3_R*, which is located between amino acid residues 922–929 and forms the optional exon encoding GDSLLDER (Fig. 3B). This alternative splice site was first reported in the insect *IP_3_R*s, but it was conserved in the human *IP_3_R1*^21^.

### Conserved structural domains in TcRyR and TcIP_3_R

Similar to the mammalian RyR and IP_3_R proteins^22^, several structural domains common to both CRCs were identified including the suppressor-domain-like domain (SD), MIR (Mannosyltransferase, IP_3_R and RyR) domain, two RIH (RyR and IP_3_R Homology) domains, and an RIH–associated (RIHA) domain. The sequence identities between these common domains of TcRyR and TcIP_3_R range from 14.6% to 25.4% (Table 2). Additionally, six transmembrane helices (TM1 to TM6) were predicted in the COOH-terminal region of both TcRyR (4438–4460,4624–4646,4701–4723,4843–4865,4891–4913, 4971–4990) and TcIP_3_R (2266–2288, 2295–2317, 2343–2365,2386–2408, 2431–2453, 2546–2568). The GGGXGD motif between TM5 and TM6 that acts as the selectivity filter was also conserved in TcRyR (4947–4952) and TcIP_3_R (2521–2526). Like mammalian RyRs, three copies of a repeat termed SPRY (SPla and RyR) domain (659–795, 1084–1205, 1540–1680) and four copies of a repeat termed RyR domain(846–940, 959–1053, 2826–2919, 2942–3030) were also predicted in TcRyR.

### Developmental expression of *TcRyR* and *TcIP_3_R*

**Figure 4:**
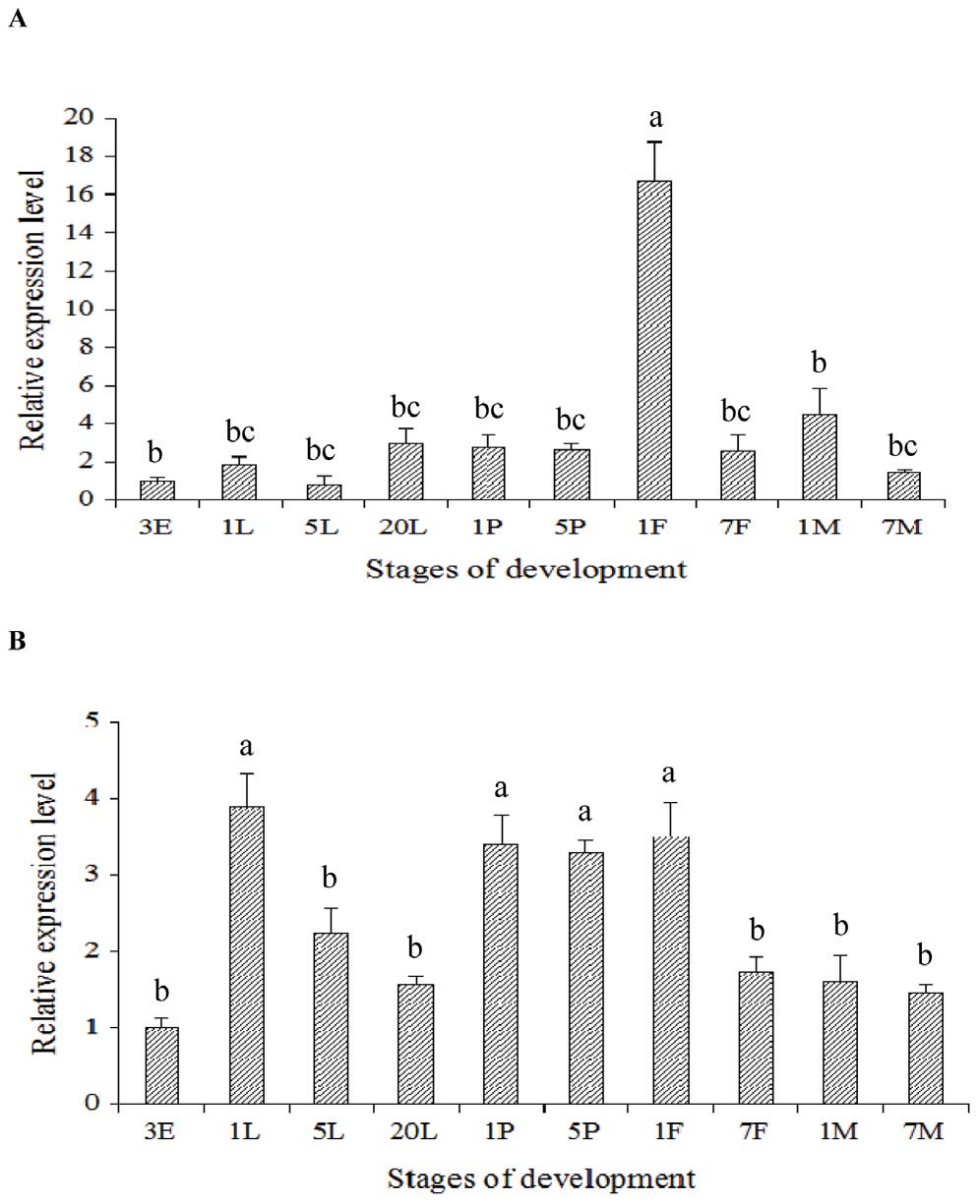
Relative mRNA expression levels of *TcRyR* (A) and *TcIP_3_R* (B) in the different development stages of *Tribolium castaneum*. The relative expression level was expressed as the mean ± SE (N = 3), with the 3-day old egg as the calibrator. The different lowercase letters above the columns indicate significant differences at the P<0.05 level. 3E: 3-day-old egg; 1L: 1-day-old larvae; 5L: 5-day-old larvae; 20L: 20-day-old larvae;1P: 1- day-old pupa; 5P: 5-day-old pupa; 1F: 1-day-old female adult; 7F: 7-day-old female adult; 1M: 1-day-old male adult; 7M: 7-day-old male adult.

To gain understanding of the developmental expression of *TcRyR* and *TcIP_3_R* in *T. castanuem*, the mRNA levels of these two CRC genes were analyzed using RT-qPCR at different developmental stages of *T. castanuem* insects, including 3-day-old eggs, 1-, 5- and 20-day-old larvae, 1- and 5-day-old pupae, 1- and 7-day-old female adults, and 1- and 7-day-old male adults. The developmental expression pattern revealed that the mRNA levels of *TcRyR* were highest in the 1-day-old female adults, while there was no significant difference among the egg, larval and pupal stages (Fig. 4A). The highest and lowest mRNA expression levels of *TcIP_3_R* were observed in the 1-day-old larvae and 3-day-old eggs, respectively (Fig. 4B).

### RNAi of *TcRyR* and *TcIP_3_R*

**Figure 5:**
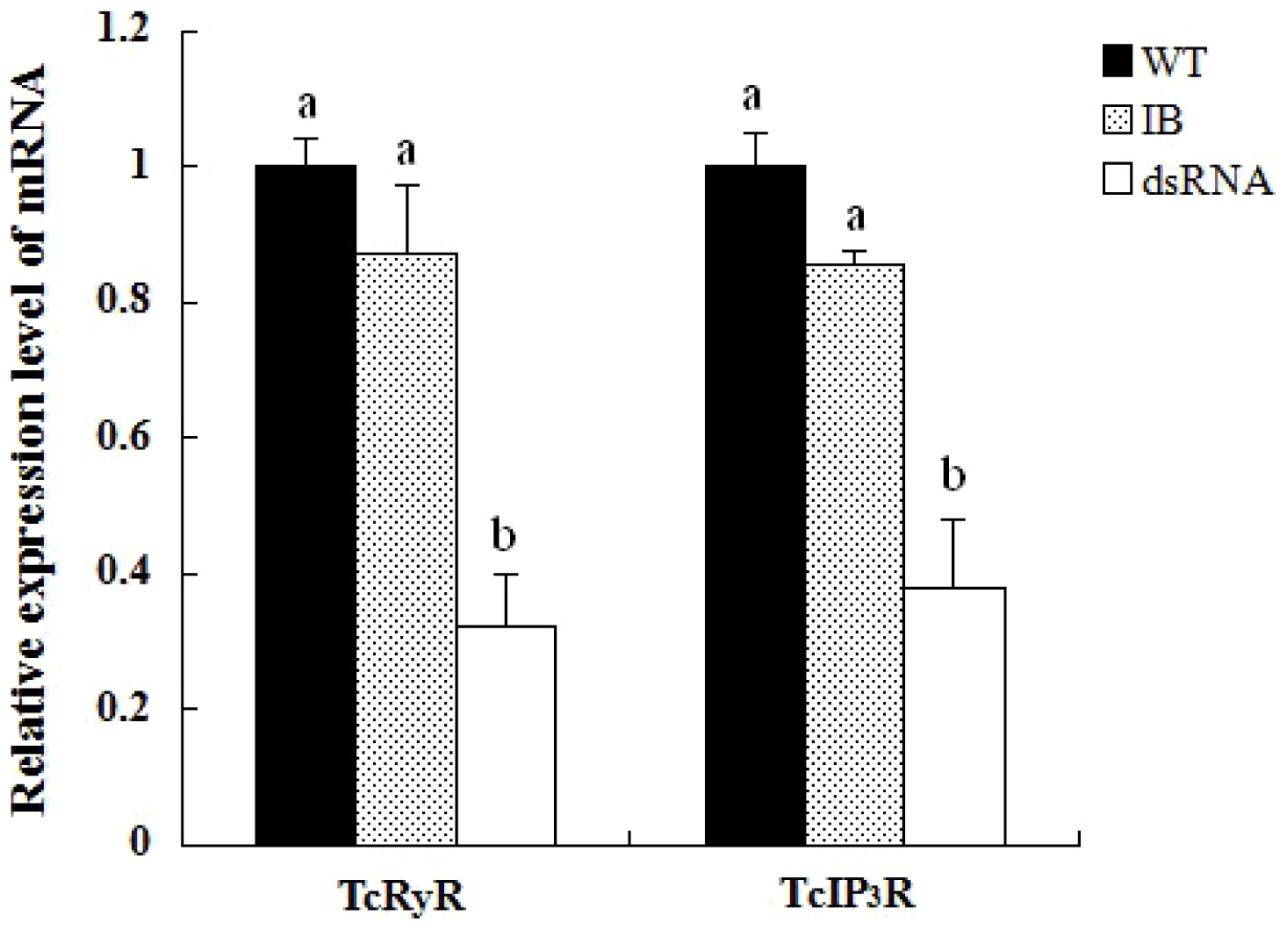
Expressions of *TcRyR* and *TcIP_3_R* transcripts in the uninjected wild-type larvae (WT group), the buffer-injected larvae (IB group), and the dsRNA injected group.

**Figure 6:**
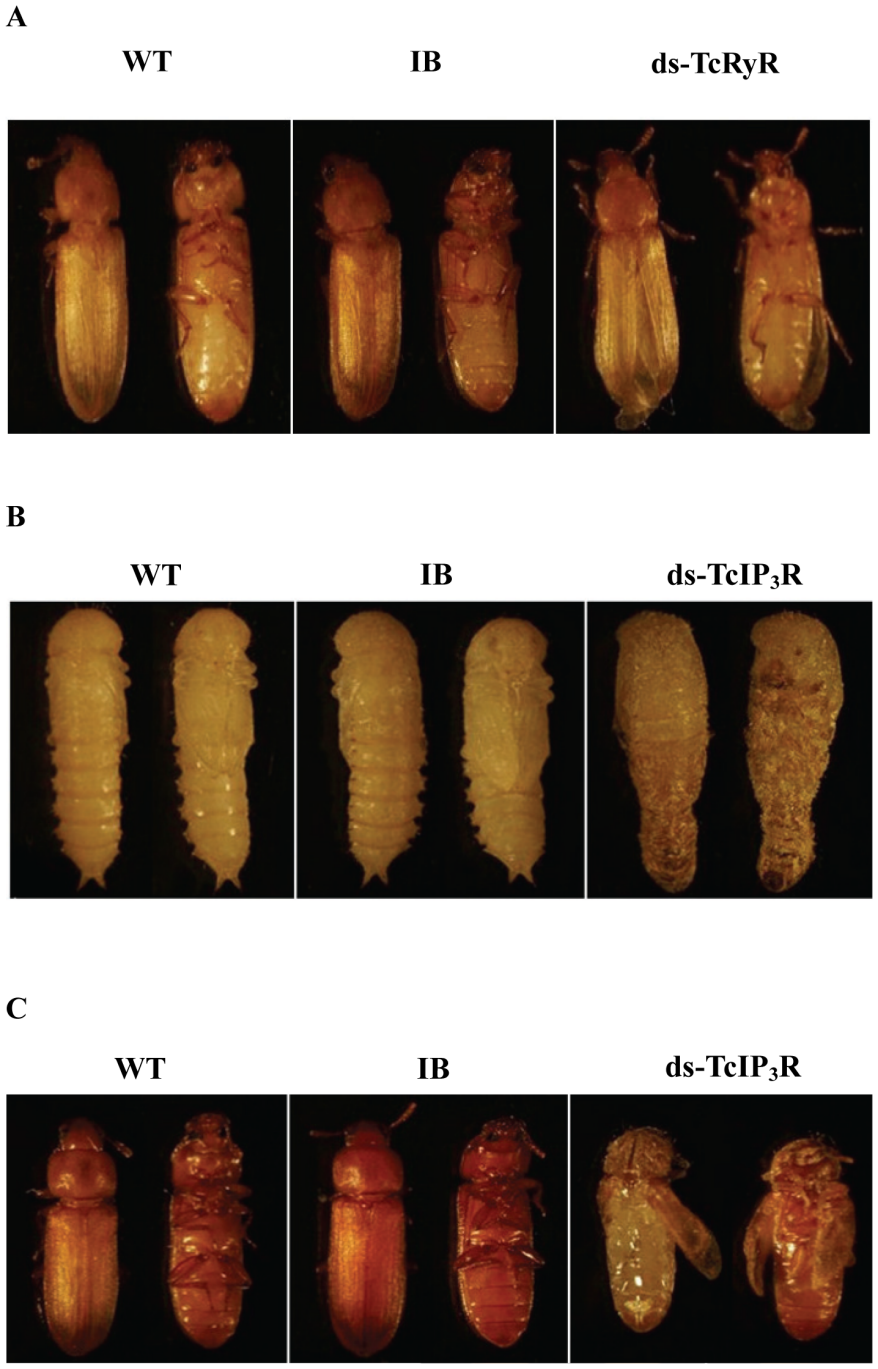
RNAi phenotypes of *TcRyR* and *TcIP_3_R*. A. Injection of dsRNA for *TcRyR* resulted in abnormal folding of the adult hind wings. B. Injection of dsRNA for *TcIP_3_R* resulted in defective larval–pupal metamorphosis. C. Injection of dsRNA for *TcIP_3_R* resulted in defective pupal-adult metamorphosis.

We employed RNAi to investigate the putative function of *TcRyR* and *TcIP_3_R.* The silencing effects of dsTcRyR and dsTcIP_3_R were detected by qPCR on the sixth day after the dsRNA injection. The results showed that the transcript levels of *TcRyR* and *TcIP_3_R* in the injected larvae were significantly suppressed by 67.86% and 61.99%, respectively, compared with those in the uninjected wild-type larvae (Fig. 5). While the injected larvae with dsTcRyR underwent normal larval–larval and larval–pupal molts and developed into adults, the hind wings of 65.9% of the individual adults could not fold properly (Fig. 6A), and all individual adults lost their ability to crawl early in adulthood and died two weeks later. In the group treated with dsTcIP_3_R, 64.7% of the larvae were unable to cast their molts completely and could not undergo normal larval–pupal metamorphosis (Fig. 6B), and thus died entrapped in their larval cuticles during the pupal stage. While the rest of the larvae could develop into pupae, the pupae could not undergo normal pupal-adult metamorphosis (Fig. 6C).

## Discussion

Developing insecticides that act on novel biochemical targets is important for crop protection due to the ability of insects to rapidly evolve insecticide resistance. It has been suggested that insect calcium channels would offer an excellent insecticide target for commercial exploitation^23^,^24^, and the recent discovery of diamide insecticides has prompted the studies on insect RyRs. However, no insecticidal compounds targeting IP_3_Rs have been reported so far in the literature, and the studies on insect IP_3_R are solely limited to *Drosophila*. In this study, we cloned and characterized RyR and IP_3_R genes from *T. castaneum.* As with other invertebrates, the sequencing data evidenced the existence of only a single RyR and IP_3_R gene, *TcRyR* and *TcIP_3_R*, in *T. castaneum*, which was supported by homology searches on the *T. castaneum* genomic database. The amino acid identities of TcRyR with human homologues (44–46%) were considerably lower than those observed with TcIP_3_R (53%–61%), which may suggest that RyRs are better targets for insecticidal molecules with lower mammalian toxicity.

Despite the large difference in size and the low amino acid identity between *TcRyR* and *TcIP_3_R*, these two CRCs share a similar architecture consisting of NH_2_-terminal modular regulatory domains that contain an RIH-RIH-RIHA arrangement and a COOH-terminal transmembrane (TM) domain that contains the conserved GGGXGD motif. The RIH-RIH-RIHA arrangement is also found in many “ancestral CRC” eukaryotic proteins, but it is undetectable in any prokaryotic protein^25^. In both RyRs and IP_3_Rs, the conserved GGGXGD motif acts as the selectivity filter, which enables the channels to discriminate between ions. Mutagenesis of residues in this region of both RyR and IP_3_R alters the channel conductance^26^,^27^,^28^. Recently, it was found that an IP_3_R in which the COOH-terminal transmembrane region was replaced with that from the RyR1 was blocked by ryanodine, indicating that activation mechanisms were conserved between IP_3_R and RyR^29^. These conserved structural features and activation mechanisms suggested that an ancient duplication event probably gave rise to these two classes of intracellular CRC genes. A recent study revealed that RyRs might arise from pre-existing, ancestral IP_3_R-like channels present in prokaryotes by incorporating promiscuous ‘RyR’ and ‘SPRY’ domains via horizontal gene transfer^25^.

Both RyRs and IP_3_Rs contribute to Ca^2+^ signals and play important roles in a vast array of physiological processes, as has been investigated in knockout mouse models. RyR1 knockout mice die perinatally due to respiratory failure caused by defective excitation-contraction (E–C) coupling in the diaphragm^30^, and RyR2 knockout mice died at approximately embryonic day 10 with morphological abnormalities in the heart tube^31^. In contrast, RyR3 knockout mice are viable but exhibited impairments in memory functions and social interaction^32^,^33^,^34^. IP_3_R-knockout studies have revealed that IP_3_R1-deficient mice die in utero or by the weaning period, and the survivors have severe behavioral abnormalities in the form of ataxia and epileptic seizures^35^, whereas IP_3_R2 and IP_3_R3 double knock-out mice exhibit hypoglycemia and deficits of olfactory mucus secretion, suggesting that these two isoforms play key roles in the exocrine physiology and perception of odors^36^,^37^.

While knockout studies of the mammalian RyR and IP_3_R have demonstrated their critical role in development and physiology, the functional characterization of the insect RyR and IP_3_R is still limited. In this study, the contribution of *TcRyR* and *TcIP_3_R* to the developmental and physiological outcomes was assessed by in vivo RNAi. Our data show that the suppression of the *TcRyR* transcript in the late larval stage leads to abnormalities in the folding of the hind wings and crawling behavior in adults. It has been reported in the neopterous insects that the third axillary sclerite and muscle were involved in the folding of the wing into the rest position^38^,^39^. On the other hand, it has been reported in *Drosophila* that crawling movements were powered by muscle contractions^40^. Thus, the abnormal phenotype observed in this study might be due to the impairment of muscle EC-coupling. This is consistent with previous findings showing that mutant fruit flies lacking *RYR* expression (Ryr^16^) display impairment of muscle EC-coupling in the larval development^41^, and mutant *Caenorhabditis elegans* with a defective RyR gene (*unc-68*) exhibit diminished muscle function and decreased movement^42^. On the other hand, when the mRNA expression levels of *TcIP_3_R* were suppressed in the late larval stage, defects were observed in larval–pupal and pupal-adult metamorphosis. A similar result was also observed in *Drosophila* that disruption of the *Drosophila* *IP_3_R* gene leads to lowered levels of ecdysone and delayed larval molting^43^. These results suggest that calcium release via *IP_3_R* might play an important role in regulating ecdysone synthesis and release during molting and metamorphosis in insects*.* Further research is needed to confirm this hypothesis.

## Methods

### Insects

The Georgia-1 (GA-1) strain of *T. castaneum* was cultured on 5% (w/w) yeasted flour at 30°C and 40% RH under standard Conditions^44^.

### Total RNA isolation and reverse transcription

Total RNAs were extracted using the SV total RNA isolation system (Promega, Madison, WI) according to the manufacturer's instructions. First-strand cDNA was synthesized from 5 µg of total RNA using the Primescript™ First-Strand cDNA Synthesis kit (TaKaRa, Dalian, China), according to the manufacturer's instructions.

### Polymerase chain reaction

The amino acid sequences of RyR and IP_3_R from *D. melanogaster* (GenBank: BAA41471 and AAN13240) were searched against BeetleBase (http://www.bioinformatics.ksu.edu/blast/bblast.html), and the regions with significant hits were manually annotated to identify the putative transcript and translation products. The ClustalW algorithm^45^ was used to align protein sequences to further support annotation predictions. Specific primer pairs were designed based on the sequences identified above (Table 1). PCR reactions were performed with LA Taq™ DNA polymerase (TaKaRa, Dalian, China).

### Reverse transcription quantitative PCR (RT-qPCR)

RT-qPCR reactions were performed on the Bio-Rad CFX 96 Real-time PCR system using SYBR® PrimeScript™ RT-PCR Kit II (Takara, Dalian, China) and gene specific primers (Table 1). The procedures for RT-qPCR were the same as those described by Zhu et al^46^. Ribosomal protein S3 (rps3, GenBank: CB335975) was used as an internal control^47^. The PCR reaction volume was 20 µL containing 2 µL of diluted cDNA, 0.4 μM of each primer, 10.0 µL SYBR Premix EX Taq^TM^ II(2×)and 0.4 µLROX Reference Dye II(50×). Two types of negative controls were set up including a no-template control and a reverse transcription negative control. Thermocycling conditions were set as an initial incubation of 95°C for 30 s and 40 cycles of 95°C for 10 s and 60°C for 15 s. Afterwards, a dissociation protocol with a gradient from 57°C to 95°C was used for each primer pair to verify the specificity of the RT-qPCR reaction and the absence of primer dimer. The mRNA levels were normalized to rps3 with the ΔΔC_T_ method using Bio-Rad CFX Manager 2.1 software. The means and standard errors for each time point were obtained from the average of three independent sample sets.

### Cloning and sequence analysis

RT-PCR products were cloned into the pMD18-T vector (TaKaRa, Dalian, China) and sequenced. Nucleotide sequences from individual clones were assembled into a full-length contig using the ContigExpress program, which is part of the Vector NTI Advance 9.1.0 (Carlsbad, CA*,* Invitrogen) suite of programs. The sequence alignment was performed using ClustalW^45^ with the default settings. Transmembrane region predictions were made using the TMHMM Server v.2.0 (http://www.cbs.dtu.dk/services/TMHMM/). Conserved domains were predicted using the Conserved Domains Database (NCBI) or by alignment to other published RyRs and IP_3_Rs.

### RNAi

Double-stranded RNAs (dsRNAs) were synthesized using the MEGAclear™ Kit (Ambion, Austin, TX) based on nucleotides 502-1118 (617 bp) and 1136-1646 (511 bp) of the ORF region of the *TcRyR* and *TcIP_3_R*, respectively. Each 20-day-old larva was injected with 200 nL of a solution containing approximately 200 ng of dsRNA. On the sixth day after the dsRNA injection, the insects were used to detect the suppression of the *TcRyR* and *TcIP_3_R* transcript by RT-qPCR. Afterwards, the insects were reared under the standard conditions mentioned above, and the phenotypes were visually observed. The buffer-injected larvae (IB group) and the uninjected wild-type larvae (WT group) were set as controls in all injection experiments. Three replications were carried out with at least 30 insects in each control or treatment.

### Database entries

The entire coding sequences of *TcRyR* and *TcIP_3_R* have been deposited in the GenBank and the accession numbers are KM216386 and KM216387, respectively.

## Author Contributions

Conceived and designed the experiments: J.W., B.L. Performed the experiments: Y.L., C.L., J.G., W.W., L.H., X.G. Analyzed the data: Y.L., J.W., C.L., B.L. Wrote the paper: J.W., Y.L.

## Figures and Tables

**Table 1:** Oligonucleotide primers used for RT-PCR and RT-qPCR

primer	Sequence (5′ to 3′)	Description (cDNA position)
617.TcRyRF1	AGAATGGCGGAGGCCGAAG	RyR RT-PCR product P1(1-1208)
618.TcRyRR1	ACTCTCGCAGTTCTGGATTC	
619.TcRyRF2	GACTTCAGTAGGAGTCAAGA	RyR RT-PCR product P2(1165-2569)
620.TcRyRR2	TGGAAGTGTCTACAGGGTTT	
621.TcRyRF3	GAAAGCCTCCTCCCGCAACA	RyR RT-PCR product P3(2434-2855)
622.TcRyRR3	ATTCGCCTTCTCCATAGTCT	
623.TcRyRF4	GGTTCAGACAGTCCTCCGTG	RyR RT-PCR product P4(2790-5356)
624.TcRyRR4	AGGCGGCATAAAGAGTCAAA	
625.TcRyRF5	CGCTGTATTGATGTATTGGA	RyR RT-PCR product P5(5275-6654)
626.TcRyRR5	CCACATTTCGGCAACATCTT	
627.TcRyRF6	GTACAATTTCATAAACGCCG	RyR RT-PCR product P6(6381-8034)
628.TcRyRR6	TCGGTAGACTGTGTGTAAAG	
629.TcRyRF7	CACTCCTCATCCAACACAGC	RyR RT-PCR product P7(7949-9416)
630.TcRyRR7	CAAAACAGGGAAGCCACCAT	
656.TcRyRF12	TTCATCCACCTTCAGTCGCA	RyR RT-PCR product P8(9222-11018)
657.TcRyRR12	TGCCAAGACATTTCGTCAGC	
633.TcRyRF9	GTTTTGATAATGCCCACAGC	RyR RT-PCR product P9(10702-12302)
634.TcRyRR9	AAACCTCCAACAGCGTCCCA	
635.TcRyRF10	GCTATCGGTGTTGCTAGTCA	RyR RT-PCR product P10(12187-13847)
636.TcRyRR10	GCACGATGACTGTAAGCACC	
637.TcRyRF11	AACCTGTTGTTACTGAACCT	RyR RT-PCR product P11(13784-15115)
638.TcRyRR11	AGTTGTGCTCTTGTTGGACG	
682.TcRyRF13	CATCGTTATTCTTCTGGCTA	RyR RT-PCR product P12(14934-15308)
683.TcRyRR13	CTGAAAGTGAATAGGAAGTG	
734.TcIP_3_RF1	CTGAAAACGCTCCAAAAACC	IP_3_R RT-PCR product P1(1-1647)
735.TcIP_3_RR1	CGTGTCTTGGGTCGTTCAGT	
736.TcIP_3_RF2	TTTGGACAACAACGGGGACG	IP_3_R RT-PCR product P2(1586-3158)
737.TcIP_3_RR2	AAAAATGCCCTCAGCTTGAC	
738.TcIP_3_RF3	TACCCGCTCGTCATGGATAC	IP_3_R RT-PCR product P3(2949-4296)
739.TcIP_3_RR3	ATGGCAGTAAACTATGACAC	
740.TcIP_3_RF4	AAGACACTGTATGGACGAGG	IP_3_R RT-PCR product P4(4175-5698)
741.TcIP_3_RR4	TCTTCTCTTAACTCGTCACT	
742.TcIP_3_RF5	CAAACAAGACGGGAAAGATT	IP_3_R RT-PCR product P5(5609-6999)
743.TcIP_3_RR5	TCCGTATGCCTGTCTCCCTA	
744.TcIP_3_RF6	CTCTTCTGGGTTAGTAGTTA	IP_3_R RT-PCR product P6(6795-8231)
745.TcIP_3_RR6	GACTAGGACAAGTTATCAAC	
800.Tcrps3F1	ACCGTCGTATTCGTGAATTGAC	RT-qPCR
801.Tcrps3R1	ACCTCGATACACCATAGCAAGC	
806.TcRyRF	AAGGGGTATCCTGATTTGGG	RT-qPCR (7198-7400)
807.TcRyRR	TTCGCATCTACGATAGCACG	
808.TcIP_3_RF	GTGACTTGAGCCAGGCTTTC	RT-qPCR (5491-5621)
809.TcIP_3_RR	CCCGTCTTGTTTGTCCTCAT	

**Table 2:** Percentage of amino acid sequence identity between the conserved domains of TcRyR and TcIP_3_R

	SD	MIR	RIH	RIHA
TcRyR	11-200	211-390	437-642/2218-2448	3979-4104
TcIP_3_R	5-228	236-424	463-667/1196-1376	1946-2061
identity	21.8	14.6	18.8/15.1	25.4
